# Supplementary material for: Bioinformatic Characterization and Molecular Evolution of the Lucina pectinata Hemoglobins
Source: Genes (Basel). 2022 Nov 5;13(11):2041. doi: 10.3390/genes13112041 (PMC9690805; doi:10.3390/genes13112041)
Supplement: Supplementary file 1 [file genes-13-02041-s001.zip › genes-1939546-supplementary.pdf]

Supplementary Table S1 List of organism and globins used for MSA and abbreviations used for analysis.

Organism	Class	Description	Similarity %	E-value	GenBank Accession number
**Summary of BLAST search results obtained with HbI amino acid sequence AAG01380.1 as query**					
Lucina pectinata	Bivalvia	Hemoglobin I	100	9.0E-100	P41260.4
Lucina pectinata	Bivalvia	Hemoglobin II	32	1.00E-18	AAO89499.1
Lucina pectinata	Bivalvia	Hemoglobin III	33	4.00E-16	ABS87592.1
Calyptogena nautilei	Bivalvia	Hemoglobin III	29	6.00E-12	BAD34605.1
Aplysia limacina	Gastropoda	Myoglobin	29	6.00E-10	1MBA_A
Yoldia eightsi	Bivalvia	Hemoglobin I	32	4.00E-10	Q8T7J9.1
Calyptogena soyoae	Bivalvia	Hemoglobin II	27	2.00E-10	BAD34604.1
Calyptogena tsubasa	Bivalvia	Hemoglobin II	29	1.00E-10	BAD35021.1
Calyptogena kaikoi	Bivalvia	Hemoglobin II	28	1.00E-10	BAD34602.1
Aplysia kurodai	Gastropoda	Myoglobin	26	2.00E-09	P02211.4
Scapharca inaequivalvis	Bivalvia	Hemoglobin IIA	30	3.00E-07	P14821.2
Scapharca kagoshimensis	Bivalvia	Hemoglobin (HbIIA)	30	3.00E-07	ACN90947.1
Calyptogena kaikoi	Bivalvia	Hemoglobin I	29	1.00E-07	BAD34601.1
Calyptogena soyoae	Bivalvia	Hemoglobin I	29	3.00E-06	BAD34603.1
Tegillarca granosa	Bivalvia	Hemoglobin (HbIIA)	32	2.00E-06	ADZ52803.1
**Summary of BLAST search results obtained with HbII amino acid sequence AAO89499.1 as query**					
Lucina pectinata	Bivalvia	Hemoglobin II	100	1.00E-112	AAO89499.1
Lucina pectinata	Bivalvia	Hemoglobin III	65	7.00E-72	ABS87592.1
Lucina pectinata	Bivalvia	Hemoglobin I	32	1.00E-18	P41260.4
Scapharca inaequivalvis	Bivalvia	Hemoglobin IIB	31	6.00E-07	AAB51308.1
Barbatia virescens	Bivalvia	Hemoglobin II	28	2.00E-10	BAA09587.1
Tegillarca granosa	Bivalvia	Hemoglobin (HbI)	32	1.00E-10	AEL88623.1
Scapharca broughtonii	Bivalvia	Hemoglobin	30	8.00E-09	P02212.1
Aplysia juliana	Gastropoda	Myoglobin	25	7.00E-09	BAA19794.1
Calyptogena nautilei	Bivalvia	Hemoglobin IV	31	3.00E-09	BAD34606.1
Scapharca inaequivalvis	Bivalvia	Homodimeric hemoglobin	30	3.00E-08	CAA50510.1
Scapharca kagoshimensis	Bivalvia	hemoglobin(HbI)	30	2.00E-08	ACN90946.1
Anadara trapezia	Bivalvia	Globin	33	1.00E-08	P25165.2
Calyptogena tsubasa	Bivalvia	Hemoglobin I	27	8.00E-07	BAD35020.1
Barbatia lima	Bivalvia	α chain of the tetra-meric hemoglobin	27	2.00E-07	BAA09966.1
Barbatia virescens	Bivalvia	Hemoglobin I	26	1.00E-07	AAB24577.2
Anadara trapezia	Bivalvia	Beta globin	30	3.00E-06	P04251.3
Barbatia lima	Bivalvia	Homodimeric hemoglobin δ chain	26	1.00E-05	BAA09965.1
Barbatia lima	Bivalvia	β chain of the tetra-meric hemoglobin	24	4.00E-04	BAA09967.1
**Summary of BLAST search results obtained with HbIII amino acid sequence ABS87592.1 as query**					
Lucina pectinata	Bivalvia	Hemoglobin III	100	5.00E-114	ABS87592.1
Lucina pectinata	Bivalvia	Hemoglobin II	65	7.00E-72	AAO89499.1
Lucina pectinata	Bivalvia	Hemoglobin I	33	4.00E-16	P41260.4
Spisula solidissima	Bivalvia	Nerve hemoglobin	30	9.00E-26	CAJ31107.1
Scapharca kagoshimensis	Bivalvia	Hemoglobin (HbIIB)	28	5.00E-08	ACN90948.1

A

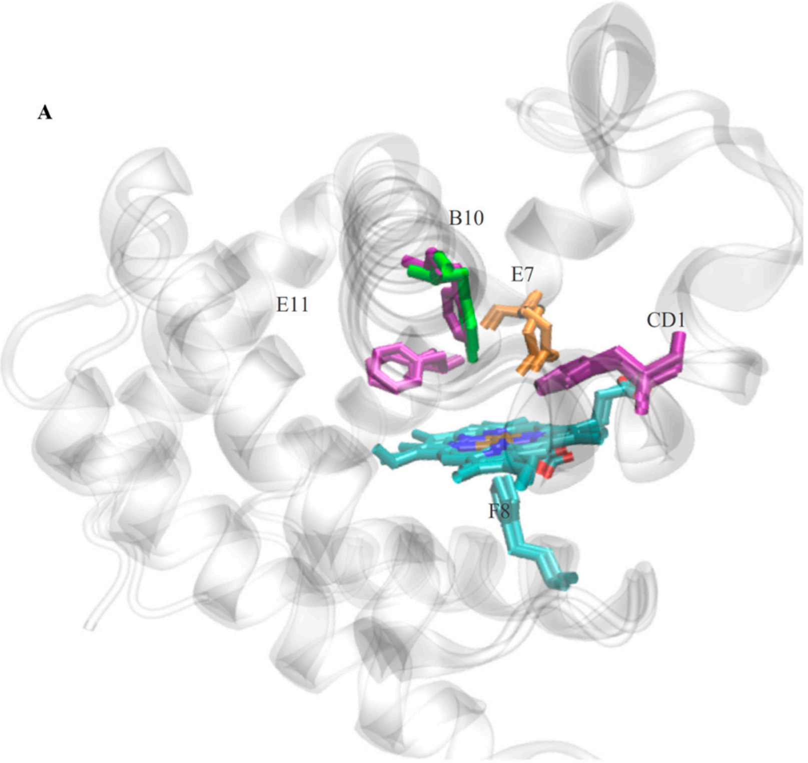

B

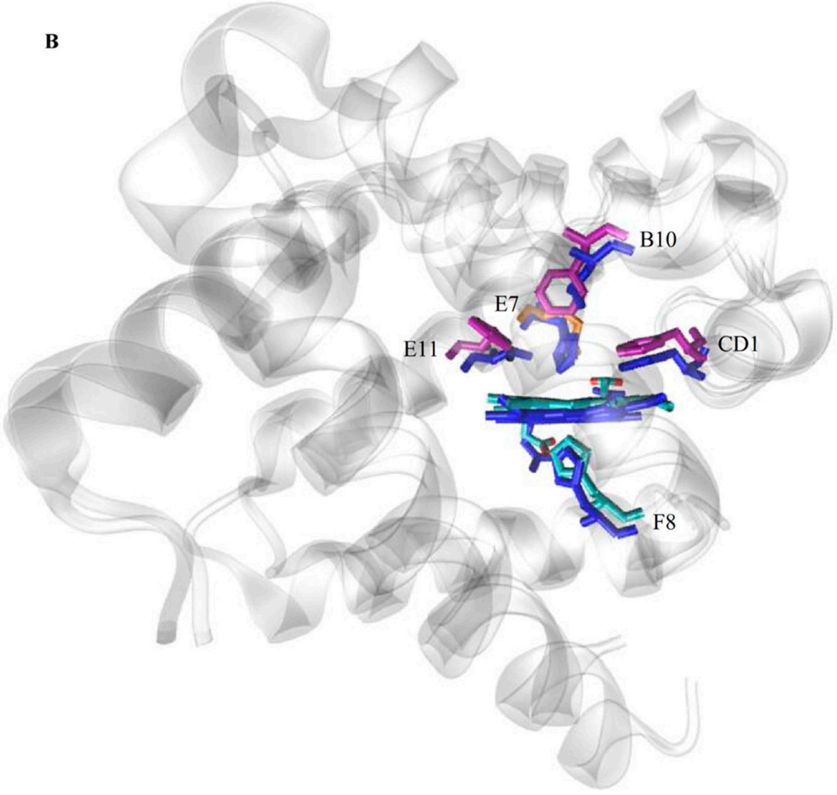

Supplementary Figure S1. A) Structural alignment of HbI_{Lp} protein structure (PDB 1FLP) and $HbII_{Lp}$ (PDB 2OLP) showing their amino acid distribution in the heme pocket. Alignment was done using the STAMP algorithm as implemented in MultiSeq in VMD. The only difference between the two heme pockets is the amino acid in

position B10, a phenylalanine for HbI_{Lp} and a tyrosine for $HbII_{Lp}$. Both hemoglobins have a phenylalanine at positions CD1 and E11, a glutamine at position E7 and a histidine at position F8. $HbII_{Lp}$ and $HbIII_{Lp}$ have the same heme pocket amino acid distribution. B) Structural alignment of HbI_{Lp} protein structure (PDB 1FLP) and Sperm Whale Myoglobin (SWM) (PDB 1A6M) showing their amino acid distribution in the heme pocket. Amino acid residues of heme pocket of SWM are colored in blue. For SWM there is a leucine at position B10, a histidine at position E7 and a valine in position E11.

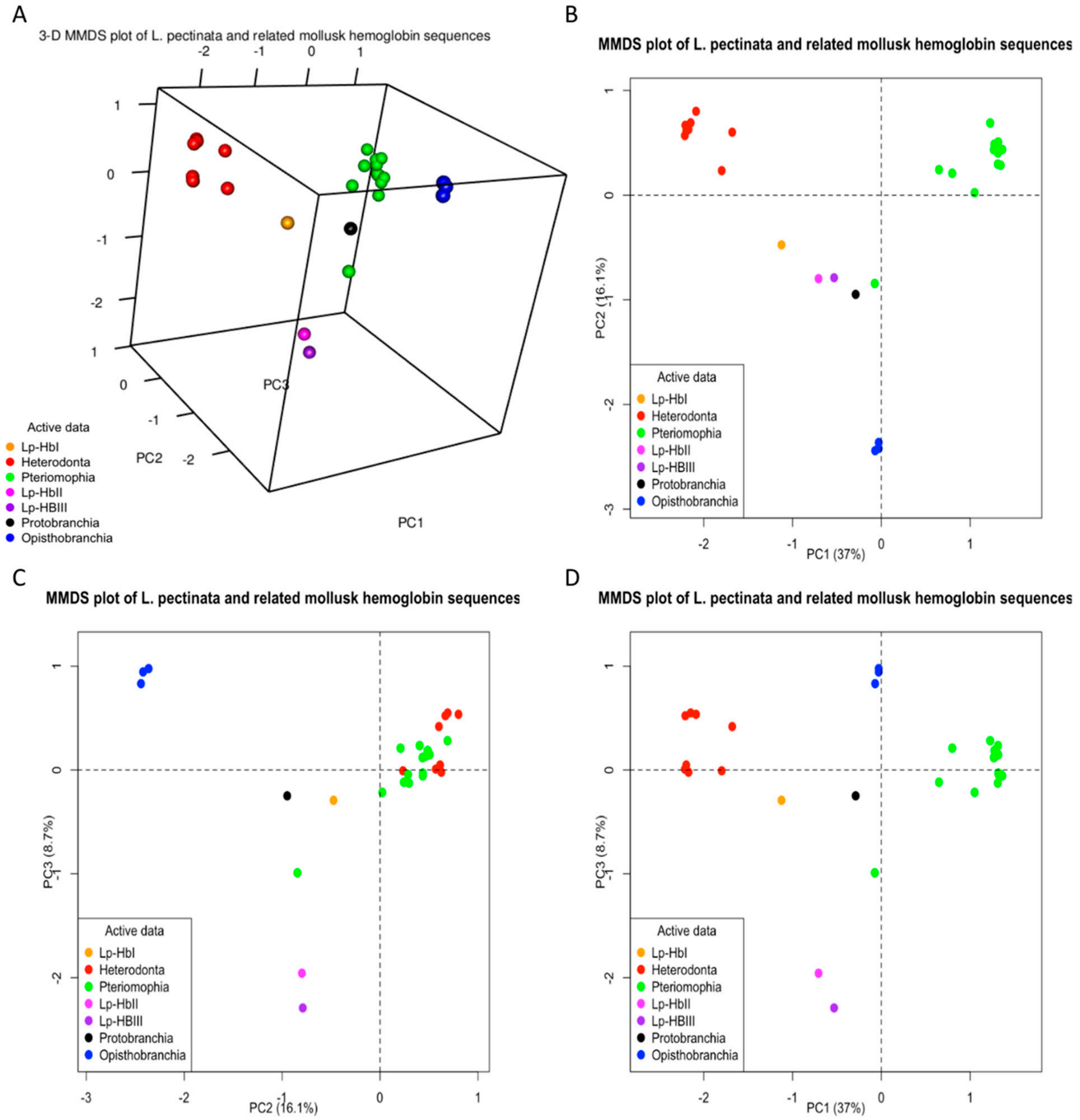

Supplementary Figure S2. A) 3D representation of the mollusk globins sequence space. A typical multiple sequence alignment of 32 homologous mollusk globins was analyzed by MMDS, with distances based on the JTT distance matrix. The 3D space is defined by the first three components of the MDS analysis. HbI_{Lp} is colored in gold, $HbII_{Lp}$ is colored in pink and $HbIII_{Lp}$ is colored purple. The rest of the sequences are color coded according to mollusk sub-families they belong to: colored in red are Heterodonta subclass, in green are the Pteriomophia subclass, in blue are the Opisthobranchia subclass, and in black the Bivalve, Y. eightsi, member of the Protobranchia subclass. **B) The 2D sequence space of mollusk globin formed by PC1 and PC2. C) The 2D sequence space of mollusk globin formed by PC3 and PC2. D) The 2D sequence space of mollusk globin formed by PC1 and PC3.** We show the 3-D projection of the first three PC. It is highly informative about the evolution and drift of these proteins. The 2D projections for pairs of these three PC are also shown in Supplementary Figure 2 as B, C and D. In this analysis we observed, as expected, that these globins are also clustered according to their subfamilies. The 2D

sequence space of PC1 and PC2, Supplementary Figure 2B, resembles the unrooted phylogenetic tree previously shown. However, the 3D sequence space clearly shows how $HbII_{Lp}$ and $HbIII_{Lp}$ are close to each other and separated from the other members of the Heterodonta subfamily. This can also be seen in the other two 2D sequence space shown in Supplementary Figure 2C and Supplementary Figure 2D. This data shows that $HbII_{Lp}$ and $HbIII_{Lp}$ have drifted evolutionarily in the sequence space.
